# Towards an Open Medical School without Checkerboards during the COVID-19 Pandemic: How to Flexibly Self-Manage General Surgery Practices in Hospitals?

**DOI:** 10.3390/healthcare9060743

**Published:** 2021-06-17

**Authors:** Héctor Guadalajara, Álvaro Palazón, Olatz Lopez-Fernandez, Pilar Esteban-Flores, José Miguel Garcia, Alicia Gutiérrez-Misis, Enrique Baca-García, Damián Garcia-Olmo

**Affiliations:** 1 Faculty of Medicine, Universidad Autónoma de Madrid, 28029 Madrid, Spain; a.palazon.rtm@gmail.com (Á.P.); josemiguelgarciaabajo@gmail.com (J.M.G.); alicia.gutierrezm@uam.es (A.G.-M.); enrique.baca@uam.es (E.B.-G.); damian.garcia@uam.es (D.G.-O.); 2 Surgery Department, Hospital Universitario Fundación Jiménez Díaz, 28040 Madrid, Spain; pilar.esteban.f@gmail.com; 3 Psychiatry Department, Hospital Universitario Fundación Jiménez Díaz, 28040 Madrid, Spain

**Keywords:** open medical school, medical education, rotations, clerkships, general surgery practice, self-directed learning, teaching styles, educational technology, mobile application, COVID-19 pandemic

## Abstract

Background: Can we create a technological solution to flexibly self-manage undergraduate General Surgery practices within hospitals? Before the pandemic, the management of clerkships was starting to depend less on checkerboards. This study aims to explore undergraduates’ perceptions of doing rotations in teaching hospitals using different teaching styles and elicit their views regarding the options of managing practices to design a mobile app that substitutes for checkerboards. Methods: In this sequential exploratory mixed methods study, 38 semi-structured interviews at a teaching hospital were conducted. The data was used to survey 124 students doing their rotations in four teaching hospitals during the first wave of COVID-19. Results: 21 themes highlighted concerns related to the practices, the teacher involvement in the students’ education, and the students’ adaptation to clinical culture. The students reported positive perceptions concerning self-managing and organizing practices via a mobile application. However, problems emerged regarding transparency, the lack of feedback, and the need for new tools. Regarding the teaching styles, the facilitator and personal models were perceived as optimal, but the personal style had no effect on using or not using a tool. Conclusions: A mobile-learning application designed like an educational opportunities’ manager tool can probably promote self-directed learning, flexible teaching, and bidirectional assessments. However, teachers who employ a personal teaching style may not need either checkerboards or a tool. This solution supports teaching at hospitals in pandemic times without checkerboards.

## 1. Introduction

New technology platforms and mobile phone applications have revealed their great potential in healthcare situations [1,2]. Similarly, internet-based medical education has further evolved through online courses [3], which are usually offered using traditional face-to-face courses. Nevertheless, there is no consensus that the current learning technologies offer an easy route to transforming medical education while covering present needs, which seem to depend on the technology, teacher, and contextual factors [4].

Regarding the medical students, they have grown up in the digital age. Office technologies have been applied to their education, e.g., emails and office software [5], and specific learning technologies such as e-learning platforms and simulations [6] have been included. Consequently, the students rely on mobile technologies and apps to manage both their daily organization and their academic training in current Higher Education Institutions (HEIs [7,8]). However, since 1995, medical education advances in the introduction of technologies in the educational processes have not increased in line with other fields of study, although they have progressively been included in clinical practice, e.g., virtual patient cases and technology-enhanced simulation [6,9]. Interestingly, challenges still exist such as the networking strategies and platforms in medical education within teaching hospitals. Thus, gaps remain and there is a need to go beyond the static and rigid educational instruction managed by checkerboards, to one that flexibly and fluidly supports medical education in these dynamic health care settings.

Furthermore, there is an overriding need for HEIs to take advantage of improving their quality and capacity to adapt to current challenges using apps [10]. Indeed, redesigning medical education for mobile learning, i.e., m-learning, is an urgent commitment due to the situation caused by the coronavirus disease 2019 (COVID-19). Public health measures prevail in Teaching Hospitals (THs). According to Rose [11], the pandemic has affected clerkship learning environments because the students may either contract or transmit the virus if they are asymptomatic, amongst other external factors, e.g., the need for new safety measures while learning, and diminished education opportunities. During the first wave, clinical rotations were paused and emerged to create alternative educational experiences to cover a gap identified in medical education within hospitals.

Nevertheless, studies must be undertaken to discover the problems of the medical students in hospitals, to ensure that the new proposals are consistent with proposed solutions [12,13]. For instance, Thistlethwaite and colleagues [14] found that clerkship effectiveness involved maintaining continuity in patient care, supervision, peer groups, and locations, which has required the preparation of the students and their clinical supervisors as well as the support of the universities; however, how has this been possible during COVID-19 times?

The first outcome of this pandemic crisis in medical education was the disruption of training activities in hospitals and the rapid translation to tailored and interactive virtual learning options (e.g., online didactics, mixed reality technology [15,16,17]). There were, however, a few positive outcomes, such as the different uses of technologies to protect onsite learning in hospitals with reduced groups of clinical training, clinical students interacting, and volunteering in the frontline [17,18,19]. In medicine and allied health sciences, the student-instructor interaction on site is a key factor, as interconnection enhances clinical training and translates to patient care [20,21], as happened during the first wave.

In Spain, our challenges regarding medical education are similar to those of other countries (e.g., to improve practices in hospitals, to better link graduate and specialized training [22]). Now the challenge is to face COVID-19 while protecting health professionals [23], who are in part the clinical teachers and students. Indeed, Spanish medical programs are not so different from other non-European Union programs (e.g., those of the UK or the US). The Spanish medicine degree has a couple of filters to access: (i) completing a bachelor’s degree after secondary school that requires high marks and six years of competitive university training (with surgical practices and rotations); (ii) there is a highly selective process to obtain access to the residency program (with five years of specialization in a type of surgery). In Europe, as in the US, there is a need to pay attention to the context in which patient care and training content are undertaken, and close monitoring of trainees is needed to determine when they meet standards [24]. This study may provide a global answer to advance in managing the medical practices in hospital settings flexibly.

Therefore, this study aimed to define the perceptions of fourth-year medical undergraduates in General Surgery practice at the ‘Universidad Autónoma de Madrid’ (UAM, Spain) prior to the COVID-19 pandemic. In addition, it aimed to evaluate the current rotations in TH with regard to the management, delivery, and teaching style by identifying specific problems, and designing solutions. There are four UAM THs (i.e., TH1, TH2, TH3, and TH4) which are similar in structure and functionality, but with different teaching styles. According to Grasha [25]: TH1 has a facilitator style via a self-management system where students select what activities to do to complete the rotation, promoting responsibility; TH3 uses a personal model style through a teacher who accompanies the students and continuously teaches them by example, with a paternalistic style; and the other two THs have a delegator style where there is not any guidance, only autonomous learning resources.

We intended to gather information that would help to guide the development and design of a new m-learning platform to ascertain whether promoting flexible self-organisation in surgical undergraduate rotations would be beneficial in any TH, under any teaching style and learning environment such as in a COVID-19 environment. Thus, we sought to answer the following research question: “Can we create and implement a mobile app based on students’ learning self-management to improve hospitals’ teaching experience by substituting for checkerboards?”

The present study aimed to explore medical undergraduates’ perceptions of doing rotations in teaching hospitals that use different teaching styles to elicit their views regarding the options of managing surgical practices more autonomously to design a mobile app that substitutes for a checkerboard, and to determine whether the teaching styles managed by the different teaching hospitals would have an impact on these views. This study was conducted at a point when UAM THs were starting to move checkerboards away from medical undergraduates doing practices and rotations in hospital settings. It was, therefore, hypothesized that there would be significant differences among students’ perceptions towards the THs according to their teaching styles as follows. Firstly, as student-instructor interaction is a key factor of practical medical education in hospitals, it was hypothesized that both facilitator and personal teaching styles (i.e., TH1 and TH3, respectively) would have significant positive perceptions regarding the rotations within hospitals. Secondly, it was hypothesized that all students at the surgery service would value an app to self-manage their practices, especially those who were in TH2 and TH4 (i.e., with a delegator teaching style).

## 2. Materials and Methods

### 2.1. Pilot Test

**Figure 1:**
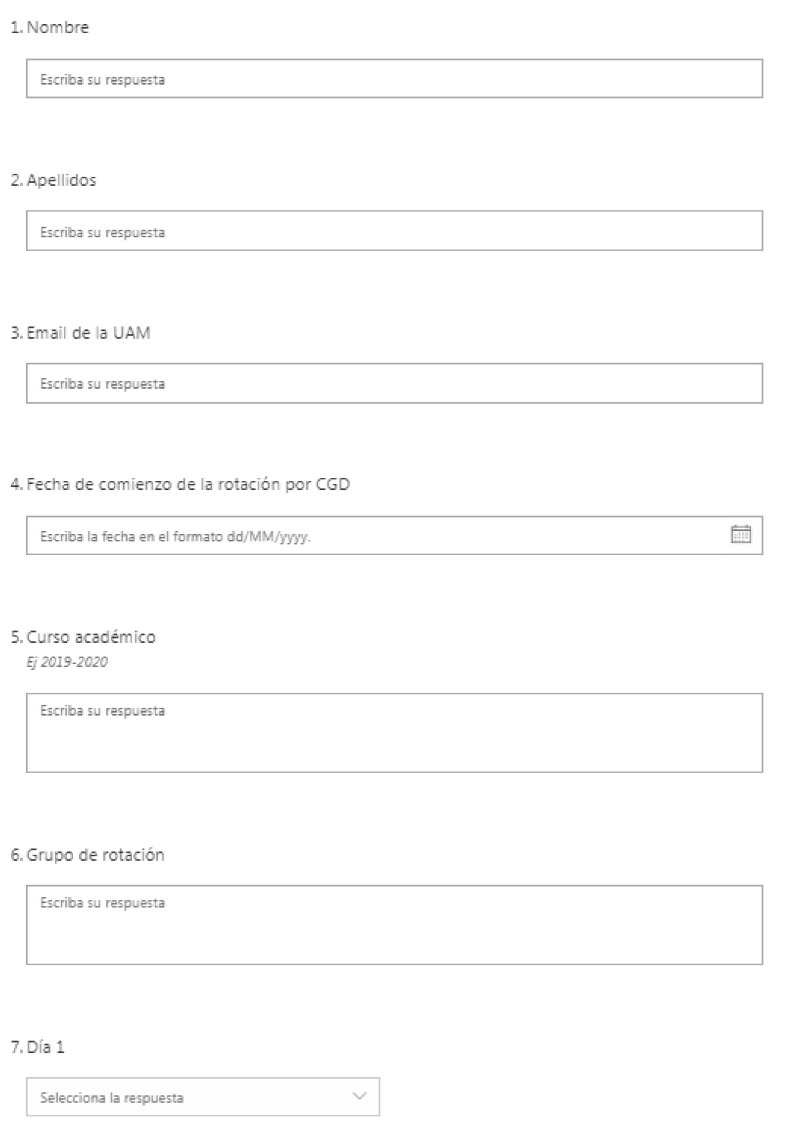
Form generated through Microsoft Office to collect the data per student related to the rotation through an excel spreadsheet to start substituting the checkerboards. Note: The names of the variables are Name, Surname, UAM email, Starting date of the rotation, Year in the degree in 2019–2020, Group of rotation, Day 1, etc.

In the first UAM Hospital (TH1), before the pandemic academic year (2018–2019), the General Surgery teaching opportunities of 4th-grade medical students were organized using Excel. This spreadsheet had a set of columns to register per student (that were in the rows) the basic information usually contained in a traditional checkerboard: name, surname, UAM email, rotation start date, year in the degree, rotation day, days per month doing the rotation in the Surgery Service, rotation signed, and rotation group. These variables were almost all generated through an online form made with Microsoft Office that included a set of instructions and codes (e.g., ‘M’ was ‘Morning shift’, ‘QF CP’ was ‘Colorectal surgery operating room [OR], ‘OBS’ was ‘clinical case’) (See Figure 1).

At the beginning of the rotation, the students were informed of the service’s teaching opportunities during their clerkship (i.e., undergraduate rotations), with a guide to the objectives they had to fulfil. Subsequently, they determined the activities they would attend. The students were able to organize their clerkship based on personal choices and availability. The proposed clerkship was collected using the described form with the activities each student proposed to carry out, indicating where, when, and what professionals they were going to need during their learning practice. At a final stage, the head of the surgery service validated the Excel spreadsheet if the students attended all the activities and then they reported signing the rotation.

### 2.2. Design

**Figure 2:**
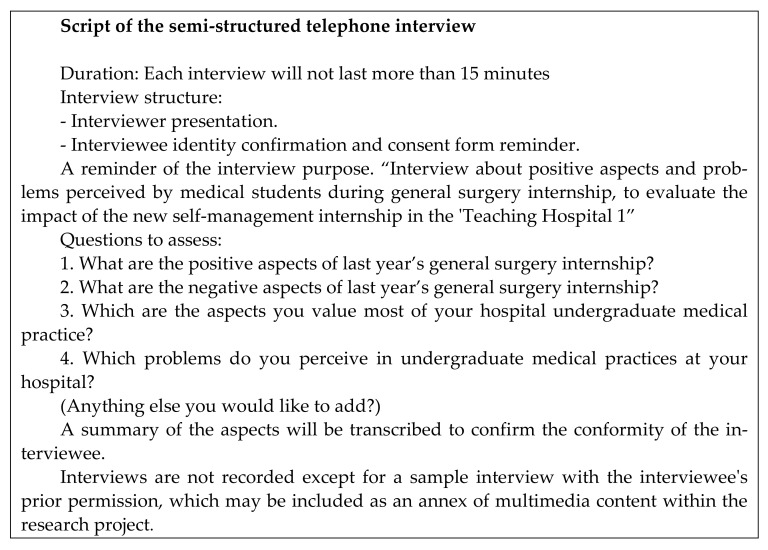
Script of the semi-structured interview used in the qualitative phase.

An exploratory sequential mixed method design (qual -> QUAN) was used for the purpose of development, in which one method informed the other method for treatment integrity [26,27,28]. This design is widely used to assess the impact of community interventions [29] using the information collected during a first qualitative phase and integrates it into a quantitative phase to develop a survey and to generalize the results [30,31]. The first qualitative phase of the study consisted of conducting semi-structured telephone interviews with volunteer students to elucidate the relevant aspects of hospital clinical clerkship experience from their point of view through a script with a set of open questions related to the experience on doing rotations in the TH1 and the possibility to self-manage them through a future mobile app (See Figure 2). In the second quantitative phase, this information was used to develop a survey to consolidate the common positive and negative rotation issues, to determine whether the teaching styles were influencing undergraduates’ educations, and to determine whether an app could solve the perceived problems detected. The design selected enabled the exploration of the heterogeneity of responses among the students in the first phase. The second phase used the information in a survey to compare the results from the THs (i.e., teaching styles) through a set of items developed through the final themes extracted from the qualitative analysis, which were measured using a Likert scale from 1–5; 1 = strongly disagree or very dissatisfied, and 5 = strongly agree or very satisfied was used.

### 2.3. Participants and Selection Criteria in Each Phase

The participants were UAM medical students in the 2019–2020 academic year. The inclusion criteria were to have carried out undergraduate surgery practices in the previous year (2018–2019) in one of the four THs associated with the UAM and not to have failed this subject. In the same way, the exclusion criteria were to have suspended the surgery practices, as well as other exceptional situations, such as being an international student doing an Erasmus stay (e.g., a European study abroad interchange financed by the European Commission), or a Spanish student doing an external rotation in at UAM THs that year.

Before the qualitative phase, the informed consent forms were provided to students to be signed if they agreed to participate in both phases of the study. In the qualitative phase, the telephone numbers of the voluntary participants doing their medical practice in the TH1 were collected in order to perform the interviews. In the quantitative phase, the email addresses of the university undergraduates who signed the consent form in the four THs were collected in order to invite them to participate in the survey.

### 2.4. Analytical Strategy in Each Phase

#### 2.4.1. Qualitative Analysis

The qualitative analysis consisted of semi-structured telephone interviews. The interviews were first transcribed as verbatim texts. A conventional qualitative content analysis was developed based on extracting themes through a labeling system that grouped the main meaning units through condensation until its saturation. The labels with more prevalence i.e., quantity, salience, and quality, were highlighted using bold letters to select the most relevant themes to include in the survey. 

#### 2.4.2. Statistical Analysis

The survey results were descriptively and psychometrically analyzed. The variable (*V*) results were shown using descriptive statistics such as means (*M*), standard deviations (*±)*, medians (*Mdn*), percentiles (*P*), and percentages (*%*) of the extreme scores (min and max). Subsequently, the validity of the survey was assessed using Exploratory Factor Analyses (EFA) through the Principal Component method (PC) with the Kaiser–Mayer–Olkin index (KMO) and Bartlett’s test of sphericity, respectively. Regarding the internal consistency, Cronbach’s alpha was estimated. In the last term, the results were compared between the THs (i.e., teaching styles) using the Kruskal–Wallis test by ranks (*H*). Any differences between the THs with *p* < 0.05 were considered significant. The data were analyzed using IBM SPSS (v 26) software.

### 2.5. Ethics

The Research Ethics Commission approved this research project of the UAM with the ID CEI 109.

## 3. Results

### 3.1. Qualitative Results from the Interviews

Thirty-eight undergraduates were interviewed by mobile telephone. Their overview was quite diverse; the students perceived the rotations at the hospital as a very heterogeneous experience with both positive and negative aspects (See Table 1). However, it was remarkable how often some themes were reiterated in most interviews, i.e., in more than 65% of the students. The most prevalent and salient ones included the organization of the practices, the involvement of the teacher in the student’s education, the adequacy of the content of the medical practice, the student involvement in clinical practice, and the self-management in organizational freedom.

### 3.2. Quantitative Results from the Surveys

Of the 247 undergraduates who attended the Gastroenterology and General Surgery course, 124 students from the four teaching hospitals (i.e., *n* _TH1_ = 31, *n* _TH2_ = 29, *n* _TH3_ = 36, *n* _TH4_ = 28) voluntarily participated in the survey. The participants responded to 20 statements selected from the previously elaborated qualitative analysis results (See Table 1), and a few additional statements about the possibility to innovate practices via a mobile app (See Table 2).

The psychometric results obtained through the EFA with the PC yielded one factor of the survey (i.e., clinical clerkship management: KMO = 0.756; Bartlett’s test: *χ*2 (190) = 847.235; *p* < 0.001) and explained 28.3% of the total variance. Concerning the internal consistency, it achieved excellent reliability for a 20-item survey, with α = 0.814 [95% CI: 0.76, 0.85].

The descriptive results were generally positive, specifically the possibility of self-managing and organizing practices with freedom (V9), the perceived adjustment between the student’s internship schedule and the hospital’s care schedule (V15), and their willingness to use a mobile app to organize their clinical clerkship. However, a few were not so positive, e.g., the statement regarding the possibility to attend the clerkship activities with enough prior academic knowledge to meet the teaching objectives (V12).

#### 3.2.1. Common Student Perceptions of the Four Teaching Hospitals

Half of the trends (45%) are common to students from all the hospitals (See Table 3). In general, in the THs (See Table 2), it is remarkable how the majority agreed that the training offered is diverse and complete (V4: P50 = 4). Meanwhile, 44% believed that there have been agglomerations that have limited their learning (V6: P50 = 3). Most of the students considered that the planning and organization of the activities was adequate (V8: P50 = 4). There was a very positive perception concerning self-management and organizational freedom during the rotations (V9: P50 = 4). However, most students disagreed with having received a refusal from professionals (V14: P50 = 2), while only 28% generally agreed. A total of 19% believed there was an adequate equity in the rotations among the hospitals (V19: P25 = 2, P50 = 3, P75 = 3, min = 1, max = 5). Interestingly, the majority agreed with the proposal of using a mobile app to organize General Surgery practice in hospitals (V20: P50 = 5), although statistical differences between the hospitals were detected in particular with regard to this last item.

#### 3.2.2. The Different Student Perceptions of the Four Teaching Hospitals

The remaining half of the variables evaluated (55%) had significant statistical differences between the hospitals (See Table 3). Overall, a better perception from students at TH1 and TH3, i.e., facilitator and personal teaching styles, respectively, was observed, compared with TH2 and TH4 who employ the delegator teaching style. Specifically, this was regarding the relationship between the clinical teachers and the students (See: V1, V2, V10, V11, V17, V18), and assessments (See: V11, V13, V17). Interestingly, the students belonging to TH1 and TH3 perceived a better use of resources (V18). However, regarding the use of an app, those from TH3 who were taught by a personal teaching style did not express the same high level of satisfaction as the other THs where the facilitator and delegator styles are used.

Concerning the perceived problems, the main challenge detected was the ability to attend activities with sufficient prior information and knowledge to achieve their objectives (V12: P50 = 2). Subsequently, half of the students expressed the need to have had a tutor to guide the rotation and to whom they could talk (V10: P50 = 2.5); followed by 41% who were dissatisfied with the possibility of giving feedback about the practices (V17: P50 = 3), and one third who were displeased with the methods used to track attendance (V11: P50 = 3) or to evaluate clinical practices (V13: P50 = 4). In other words, the relevant components related to educational management and the methods of conducting practices within hospitals.

## 4. Discussion

The aim of the present study was to explore medical undergraduates’ perception of doing rotations in teaching hospitals that use different teaching styles to elicit their views regarding the options of managing surgical practices more autonomously to design a mobile app that substitutes for checkerboards, and value if the teaching styles managed by the different teaching hospitals would have an impact on these views.

This study was conducted when UAM THs started to move away from checkerboards from medical education in hospitals and during the beginning of the COVID-19 pandemic. In summary, the hypotheses were partly confirmed. The facilitator and personal teaching styles [25] were significantly positive regarding undergraduate perceptions on the student-instruction relationship, methodology, and care control. However, although students positively valued an app to self-manage their surgery practices, those who were in TH3 with a personal style were significantly less positive towards this outcome; it seems that for them, the close relationship with the clinical trainer was showing no need for a checkerboard or an app to self-manage their learning in the hospital. Thus, this mixed-methods study has for the first time provided the knowledge about what medical students value more and what is making them facing challenges regarding the rotations in the surgery services during Spanish medical degrees. Moreover, these qualitative themes have been transposed in a valid survey to measure this goodness and concerns in half of the enrolled students in the fourth-year medical degree doing General Surgery at UAM in 2019–2020. The findings extracted from this second phase served as a validation of the first phase. The results are quite homogeneous regarding their interest in the organization, management, and student-instructor interaction during the rotations. However, the second phase provided the finding regarding the different perceptions of the diverse teaching styles tested. The personal style seems to not affect the need for tools to manage and control practices conducted by Spanish students. In other words, the present study has provided triangulation of the findings coming from different approaches, students, and techniques [26,27,28], as well as a mutual integration of findings highlighting a novel discovery regarding the influence of a personal style in a medical speciality so unique as surgery, in which the interpersonal component is essential in its transmission and continuous practice for less coordinated groups.

Regarding the methodology of self-management, undergraduates had a heterogeneous overview. They remarked on two main factors: management, e.g., organization, self-management, content adequacy, and avoiding agglomerations; and connection, e.g., the teacher’s involvement in the student’s education, the student’s involvement within the hospital culture, or the tutor availability. Previous studies carried out in General Surgery have highlighted the critical views regarding clerkships, which constitute a stressful change due to the transition from colleges to clinical environments [32]. Wallace et al. [33] found that students described mobile devices as enabling the promotion of information, time management, and communication, as well as having several other advantages such as portability, flexibility, and accessibility. However, a few described some disadvantages such as superficial learning, distraction, and privacy concerns. Recently, Surmon et al. [34] identified similar factors that impacted on clerkships, targeting competence and disconnection as the main problems, and the need to feel part of a team, ensuring adjustments, and caring for the curriculum in hospitals. Therefore, it would appear that global trends are emerging concerning medical education in hospitals, which requires flexibility and preclinical educational strategies, e.g., by enhancing content contextualization, opportunities, and ensuring constructive alignment.

**Figure 3:**
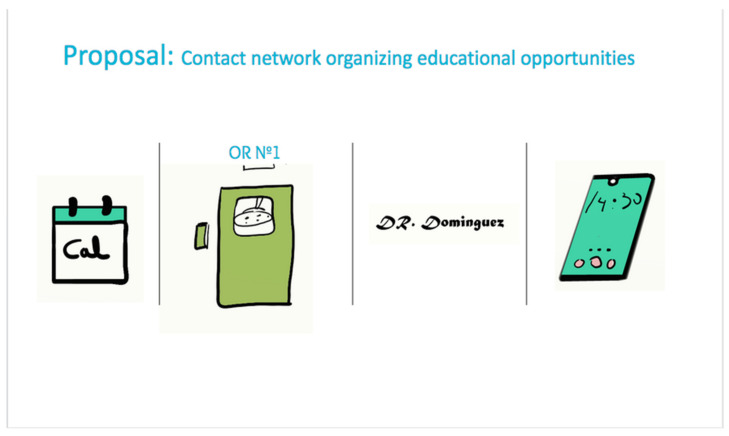
Components Included in the m-Learning App Proposed as a Medical Education Opportunities Self-Management Tool.

**Figure 4:**
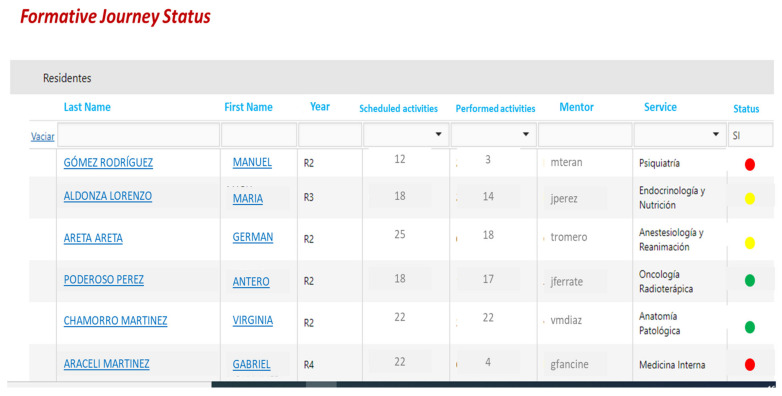
Registration of a User Training Itinerary (Screenshot). Note: Completed (green), In Progress (yellow), and None Achieved (red) goals are displayed in the final column (Status—of the activity).

Concerning the perception of the rotations, the UAM students highly valued the possibility of managing their activities through an app if an organization of rotations was ensured (See Figure 3). They requested transparency to know about the activities required to achieve the goals, and more support from the tutors, as well as options to provide feedback about the activities and other methods to track their learning or conduct assessments. Interestingly, these are specific functionalities of the app designed whereby the students can participate in the development of their rotations by selecting activities with enough information, time, and clarity involved in the methods, while they can rank the practices. As in the case of Harvard Medical School, this app promotes a collaborative model in which the students share the responsibility with their training itinerary [35] (See Figure 4).

As far as the authors are aware, currently there are not any pedagogical experiences in medical education within hospitals providing a flexible innovation such as this one, even with the pressure on medical trainees to work independently in clinical practice [36]. It can be a solution to the heterogeneity of opinions and circumstances involved in completing the rotations effectively, and could ensure that medical students perceive their needs are covered. The app can positively discriminate by providing more visibility in their activities, tracking opportunities proposed by the clinical teachers that the students can assess. This latter action covers the need for students’ feedback on their activities while palliating the perceived dissatisfaction about students’ rejection.

Another relevant challenge was the agglomerations in the OR [37]. The new app can show how many students per activity are subscribed, i.e., distance security in an OR. The app can cover online and offline activities, as surgery requires face-to-face learning (e.g., OR). Similar smartphone apps have started to be used in hospitals to improve the interaction among the educational actors [38]. Moreover, to cover the perceived need of the support of tutors on students’ learning, or the need for additional knowledge prior to the activities, didactic videos and online resources can be included in the activities [39,40,41] before doing them. This is what the flipped classroom model has shown for surgery clerkships [41,42], in which blended strategies are successful in self-directed learning. Interestingly, what initially started out as a study to investigate the educational problems in medical rotations to test and develop a solution has unexpectedly provided a clue to maintain clerkships without the use of checkerboards and with safety procedures during the COVID-19 pandemic, as other similar experiences in surgical residency programs that have also found creative, innovative, and practical solutions for the continuity of student’s surgical training and teaching (e.g., virtual educational curricula, skills development classes, video-based feedback, and simulation [41]).

Concerning the teaching styles, one of the most interesting findings is that depending on the surgery teaching style, students differed in their perception of whether a technological aid to self-manage their practices was necessary or not. Indeed, a personal model which promotes dependency seems to be equally positive to a facilitator model which enabled responsibility. However, the dependent style seems to reduce the need for an aid, while contrarily the other styles value it. The reason behind this affirmation can be related to the care that clinical instructors with a personal style can transmit to their students (i.e., through an inter-subjective and transpersonal relationship developed through interconnection of mutual respect and transformation), which as demonstrated in other health and allied sciences is essential for student education in hospitals (e.g., nursing students [43]). In surgery education, there is a lack of literature regarding teaching styles, and surgeons are encouraged to reflect upon their particular individual teaching style to provide options [44]. According to O’Brien et al. [45], existing challenges may be more complicated, as the students struggle with roles and responsibilities and adapting to frequent changes, which may require other strategies and technologies more closely associated with their social life (e.g., social networking sites).

Furthermore, in the current COVID-19 pandemic, the relationship between humans and digital technologies has rapidly evolved in healthcare and clinical education within hospitals. A current review targeting this relationship [46] has found 28 educational options used in this crisis (from computers to artificial intelligence), in eight types of users (especially medical professionals), doing 32 types of activities (e.g., providing health services remotely, differently communicating), and achieving 35 types of effects (e.g., continued education improved patient outcomes).

Thus, amid the crisis, positive outcomes such as this medical education experience have emerged as a catalyst for innovation in healthcare and the re-envisioning of medical education. However, it has also addressed the hidden curriculum of COVID-19 with its potential erosion of empathy among current medical students associated with less autonomy upon entry to the clinical arena, lack of consistent peer group preclinical years, and a training environment characterized by human suffering and death. The student-instruction interaction should be protected on-site, as it is connected to the empathetic capacity with its long-term consequences in the physician-patient relationship. There is a need for a current humanistic medicine approach in clinical and academic students training that can be developed face-to-face in the hospital in particular [47].

This study, nonetheless, has its limitations. The non-probabilistic sampling strategy and half of the undergraduates answering the survey makes it difficult to generalize the results. However, the number of interviews performed in the qualitative phase is highly considerable in comparison with similar qualitative studies, as well as the number of items included in the survey regarding the participant pool [48] that has also accomplished with an optimal level of validation. Both methods are based on self-reported measures [49], but the findings are consistent with the literature, and more importantly and unexpected is that the solution achieved to self-manage surgical practices in hospitals has fit in a critical moment where social distancing and other health and safety protocols started to emerge during the first wave of the COVID-19 pandemic. Current discussion has boosted the generation of a new educational networking manager platform that promotes continuing surgery education during the pandemic times with safety measures [11,14], which is now being tested during the second and third waves of this unprecedented pandemic.

## 5. Conclusions

To conclude, in 2010, there was a call by the Carnegie Foundation for the reformation of medical education citing the inflexibility of medical training, which they considered to be overly long and not sufficiently learner-centered [50]. This study has first tested the main concerns and benefits perceived by medical undergraduates regarding the management of rotation they were doing in THs. It then subsequently tested whether the issues extended to all UAM THs and whether a self-management tool through an app could theoretically solve the main concerns. This mixed-methods study, therefore, provided an answer, and a solution for surgery education based on the need for self-management, connection, transparency, and new methods through a new m-learning app designed as a network that can work as a teaching opportunities’ manager and can substitute checkerboards. It can promote an open self-directed medical education in hospitals with flexibility, connecting students and teachers to safely learn in clinical environments, with connectivity and both mutual and rapid assessments. Despite this fact, some teachers who teach using a personal style may not need either the checkerboards or a self-manager tool. Finally, this app can support clerkship managers to fluidly organize and prepare for unprecedented situations while teaching medical education in COVID-19’s unprecedented times.

## Figures and Tables

**Table 1:** Themes (and overall frequency), subtheme/s (and frequency), and proposed wording for the items in the survey extracted from the semi-structured telephone interviews.

Themes (Overall Frequency)	Subtheme/s (Frequency)	Wording Used in the Survey
Clerkship and rotations organization (49)	- General organization (25) - 1st day rotation organization (10) - Organizational data prior to surgery/rotation (14)	“The planning and the organization of the rotation activities have been adequate.”
Teacher involvement (40)	- Tutor involvement	“The teacher has been concerned about my learning during the rotation.”
Adequacy of clerkship content (35)	- Adequacy/Usefulness of the knowledge learned (23) - Variety of training offered (12)	“The content of the practice and the knowledge learned in it have been useful, complete and appropriate to the teaching objectives (of the guide).”
Student active participation in clinical clerkship (33)	- Student involvement in routine clinical practice (33)	“Participation in the activities of the Service/Unit during the rotation has been adequate.”
Self-management and organizational freedom (25)	- Self-management and organizational freedom (25)	“I positively value the self-management and organizational freedom allowed during the rotation.”
Tutor-student relationship (20)	- Doctor-student interaction (16) - Team/Interpersonal Environment (4)	“The doctor-student relationship has been promoted, to increase coordination and communication between both.”
Student agglomerations (18)	- Agglomeration of students	“I consider that there have been moments of student agglomeration that have limited my learning.”
Reference tutor availability (14)	- Reference teacher/tutor	“I have had a tutor or teaching person of reference to guide me and to whom I can turn.”
Attendance supervision methodology and profit of the practices (13)	- Methodology of care control (7) - Signature/time sheets (6)	“The methodology of care control and use of practices by the student has been adequate”
Prior academic knowledge related to surgery (13)	- Academic knowledge prior to surgery (8) - Access to medical records (5)	“I have been able to attend the activities of my rotation with sufficient prior academic knowledge to meet the teaching objectives.”
Assessment methodology (12)	- Evaluation methodology	“The methodology for evaluating clinical practices has been (clear) and adequate”
Student rejection (12)	- Student rejection	“I have received refusal to attend the practical activity from professionals”
Adjustment between the students’ practice schedule with the hospital clinical schedule (11)	-Concordance between the schedule of usual clinical practice and the student’s schedule	“The student’s practice schedule is adjusted appropriately to the Hospital’s care schedule.”
Adequate teaching and clinical load of the professional/tutor (8)	- Adequacy of the teaching and care load of the professional	“I perceive a teaching and care load from the professional that is adequate to receive quality care”
Clerkship time (7)	- Rotation time	“The duration of the general surgery practice rotation is adequate”
Workload (6)	- Workload	“There is an excessive workload required from students during rotation”
Knowledge of the student’s academic goals held by physicians (5)	- Knowledge of the student’s academic goals held by doctors	“There is adequate knowledge of the student’s objectives from the teaching physicians”
Medical facilities (2)	- Facilities	“The facilities used are adequate for my rotation.”
Cohesion and equity among hospitals (2)	- Cohesion/Equity among Hospitals	“There is adequate equity in the practices between the different hospitals.”
Self-knowledge of the required competencies (1)	- Self-knowledge of the required skills	“I have adequate knowledge about the skills I must obtain and that are required from me during the practices.”
Training feedback (1)	- Possibility of feedback	“The possibility of giving feedback on the practices is sufficient and adequate.”

Note: The first five themes were the most prevalent and salient themes.

**Table 2:** Overall results from the four teaching hospitals.

Items from the Survey & Responses	Very Unsatisfied	Unsatisfied	Somewhat Satisfied	Satisfied	Very Satisfied	Total
*Vx: Item*	*Fi (%)*	*Fi (%)*	*Fi (%)*	*Fi (%)*	*Fi (%)*	*M* *(±)*
V1: The doctor-student relationship has been promoted to increase coordination and communication between both.	8 (7%)	20 (16%)	42 (34%)	**40 (32%)**	**14 (11%)**	3.26 (1.07)
V2: The faculty has been concerned about my learning during the clinical clerkship.	4 (3%)	25 (20%)	46 (37%)	**32 (26%)**	**17 (14%)**	3.27 (1.04)
V3: The student’s participation in the activities of the Service during the clinical clerkship has been adequate.	11 (9%)	25 (20%)	37 (30%)	**32 (26%)**	**19 (15%)**	3.19 (1.19)
V4: The training offered has been varied and complete.	4 (3%)	11 (9%)	23 (19%)	**47 (38%)**	**39 (32%)**	**3.85 (1.07)**
V5: The content of the practice and the knowledge learned in it have been useful and appropriate to the teaching objectives.	5 (4%)	20 (16%)	37 (30%)	**40 (32%)**	**22 (18%)**	3.44 (1.08)
V6: I consider that there have been moments of agglomeration of students which have limited my learning.	15 (12%)	28 (23%)	27 (22%)	**27 (22%)**	**27 (22%)**	3.19 (1.33)
V7: The clinical clerkship plan was defined from the beginning.	12 (10%)	8 (7%)	27 (22%)	**28 (23%)**	**49 (40%)**	**3.76 (1.30)**
V8: The planning and organization of the clinical clerkship activities has been adequate.	4 (3%)	16 (13%)	27 (22%)	**51 (41%)**	**26 (21%)**	**3.64 (1.05)**
V9: I positively value the self-management and organizational freedom allowed during the clinical clerkship.	3 (2%)	6 (5%)	17 (14%)	**44 (36%)**	**54 (44%)**	**4.13 (0.99)**
V10: I have had a tutor of reference to guide me and to whom I can talk to.	**28 (23%)**	**34 (27%)**	26 (21%)	24 (19%)	12 (9%)	2.66 (1.29)
V11: The methodology of care control and use of the practices by the students has been adequate.	8 (7%)	39 (32%)	41 (33%)	25 (20%)	11 (9%)	2.94 (1.07)
V12: I have been able to attend the activities of my clerkship with sufficient prior academic knowledge to meet the teaching objectives.	**21 (17%)**	**48 (39%)**	36 (29%)	14 (11%)	5 (4%)	2.47 (1.03)
V13: The methodology for evaluating clinical practices has been clear and adequate.	13 (11%)	24 (19%)	22 (18%)	**50 (40%)**	**15 (12%)**	3.24 (1.21)
V14: I have received refusal to attend the practical activity from professionals.	**27 (22%)**	**38 (31%)**	24 (19%)	19 (15%)	16 (13%)	2.67 (1.32)
V15: The student’s internship schedule is appropriately adjusted to the hospital’s care schedule.	7 (6%)	4 (3%)	12 (10%)	**50 (40%)**	**51 (41%)**	**4.08 (1.07)**
V16: I perceive a teaching and care load from the professional that is adequate to receive quality care.	8 (7%)	23 (19%)	40 (32%)	**37 (30%)**	**16 (13%)**	3.24 (1.21)
V17: The possibility of giving feedback on the practices is sufficient and adequate.	**22 (18%)**	**29 (23%)**	28 (23%)	35 (28%)	10 (8%)	2.85 (1.24)
V18: The teaching resources of the hospital are used in a rational way.	4 (3%)	23 (19%)	33 (27%)	**39 (32%)**	**25 (20%)**	**3.47 (1.11)**
V19: There is adequate equity in the practices between the different teaching hospitals.	**22 (18%)**	**29 (23%)**	47 (38%)	23 (19%)	3 (2%)	2.65 (1.05)
V20: You would like to use a mobile app to organize your clinical clerkship in general surgery.	7 (6%)	8 (7%)	23 (19%)	**19 (15%)**	**67 (54%)**	**4.06 (1.23)**

Note: *V* = variable, x = number of the variable, item = statement, *Fi* = frequency, *%* = percentage, *M* = mean, *± =* standard deviation. The **most important findings are highlighted in bold letters**.

**Table 3:** A Comparison of the Four Teaching Hospitals.

Evaluated Variables	TH1.	TH2	TH3	TH4	*H_(3)_*	*p*
*Vx:* item	*Mdn (min–max)*	*Mdn* *(min–max)*	*Mdn* *(min–max)*	*Mdn* *(min–max)*		
*V1: The doctor-student relationship has been promoted to increase coordination and communication between both.	4 (1–5)	3 (1–4)	4 (1–5)	3 (1–5)	**10.883**	**0.012**
*V2: The faculty has been concerned about my learning during the clinical clerkship.	4 (2–5)	3 (1–5)	4 (1–5)	3 (1–4)	**19.112**	**<0.001**
V3: The student’s participation in the activities of the Service during the clinical clerkship has been adequate	3 (1–5)	3 (1–5)	3 (1–5)	3 (1–5)	4.750	0.191
V4: The training offered has been varied and complete	4 (2–5)	4 (1–5)	4 (1–5)	4 (1–5)	0.788	0.853
V5: The content of the practice and the knowledge learned in it have been useful and appropriate to the teaching objectives.	3 (2–5)	4 (1–5)	4 (1–5)	3 (1–5)	6.498	0.090
V6: I consider that there have been moments of agglomeration of students which have limited my learning.	3 (1–5)	3 (1–5)	4 (1–5)	2.5 (1–5)	2.725	0.436
*V7: The clinical clerkship plan was defined from the beginning.	3 (1–5)	4 (1–4)	4.5 (1–5)	4 (1–5)	**8.301**	**0.040**
V8: The planning and organization of the clinical clerkship activities has been adequate	4 (2–5)	4 (1–5)	4 (2–5)	3.5 (1–5)	6.374	0.095
V9: I positively value the self-management and organizational freedom allowed during the clinical clerkship	4 (1–5)	4 (1–5)	4.5 (1–5)	4 (2–5)	1.176	0.759
*V10: I have had a tutor of reference to guide me and to whom I can talk to.	3 (1–5)	2 (1–4)	3 (1–5)	2 (1–5)	**17.204**	**<0.0001**
*V11: The methodology of care control and use of the practices by the students has been adequate.	3 (1–5)	2 (1–5)	3 (2–5)	2 (1–5)	**13.242**	**0.004**
*V12: I have been able to attend the activities of my clerkship with sufficient prior academic knowledge to meet the teaching objectives.	2 (1–4)	3 (1–5)	2 (1–5)	2 (1–4)	**12.036**	**0.007**
*V13: The methodology for evaluating clinical practices has been clear and adequate	4 (2–5)	3 (1–5)	4 (1–5)	2 (1–5)	**10.500**	**0.015**
V14: I have received refusal to attend the practical activity from professionals.	2 (1–5)	3 (1–5)	2 (1–5)	2 (1–5)	1.954	0.582
*V15: The student’s internship schedule is appropriately adjusted to the hospital’s care schedule.	5 (1–5)	4 (1–5)	4 (1–5)	5 (2–5)	**7.844**	**0.049**
V16: I perceive a teaching and care load from the professional that is adequate to receive quality care.	3 (1–5)	4 (1–5)	3 (1–5)	3 (1–5)	2.606	0.456
*V17: The possibility of giving feedback on the practices is sufficient and adequate.	4 (1–5)	2 (1–5)	3 (1–5)	2 (1–5)	**13.292**	**0.004**
*V18: The teaching resources of the hospital are used in a rational way.	4 (2–5)	3 (1–5)	4 (1–5)	3 (1–5)	**9.145**	**0.027**
V19: There is adequate equity in the practices among the different teaching hospitals.	3 (1–5)	3 (1–5)	3 (1–4)	2.5 (1–5)	4.849	0.183
*V20: You would like to use a mobile app to organize your clinical clerkship in general surgery.	5 (1–5)	5 (1–5)	3 (1–5)	5 (2–5)	**12.092**	**0.007**

Note: *V* = variable, *V = variable with significant differences among hospitals, x = number of the variable, item = statement, *Mdn* = median, *min* = minimum score, *max* = maximum score, *H_(3)_* = Kruskal Wallis *H*, *p* *=* level of significance.
